# A case report of adalimumab-associated optic neuritis

**DOI:** 10.1007/s12348-011-0058-2

**Published:** 2012-01-24

**Authors:** Alice Kim, Norman Saffra

**Affiliations:** Division of Ophthalmology, Maimonides Medical Center, 902 49th Street, Brooklyn, NY 11219 USA

**Keywords:** Tumor necrosis factor, Optic neuritis, Multiple sclerosis, Adalimumab

## Abstract

**Purpose:**

To describe a case of retrobulbar optic neuritis that presented within 3 weeks of adalimumab treatment initiation.

**Methods:**

This index case was evaluated with visual field testing, brain magnetic resonance imaging, lumbar puncture, and laboratory evaluation, and treated with intravenous methylprednisolone followed by a steroid taper.

**Results:**

Our patient made a full visual recovery, but was found to have extensive T2/FLAIR foci of hyperintensities that enhanced and had restricted diffusion on magnetic resonance imaging (MRI). Six months later, these demyelinating lesions still persisted and our patient was initiated on immunomodulatory treatment.

**Conclusion:**

With the extensive burden of disease at presentation and persistence of lesions on follow-up MRI, this unusual case seems to suggest an unmasking of an underlying demyelinating process by adalimumab. The clinician should be mindful of this association and monitor for any manifestations and treat appropriately.

## Introduction

This report describes a case of adalimumab associated retrobulbar optic neuritis, of which two cases have been reported thus far in the literature [1]. Adalimumab is a recombinant monoclonal antibody that binds to the cytokine tumor necrosis factor (TNFα). Various demyelinating disorders such as optic neuritis, multiple sclerosis, transverse myelitis, and Guillain–Barré have been reported in association with TNFα antagonist therapy [2–4].

## Case report

The patient is a 42-year-old African-American female with a 20-year history of a chronic idiopathic non-granulomatous anterior uveitis. Her medical history includes hypertension and genital and oral herpes, for which she was maintained on valcyclovir. Her previous evaluations for the uveitis included non-reactive FTA-ABS and RPR, negative tuberculin skin test, negative serologies of ANCA, ANA, anti-double stranded DNA, rheumatoid factor, Lyme EIA, and normal ACE level, gallium scan and chest X-ray.

**Fig. 1:**
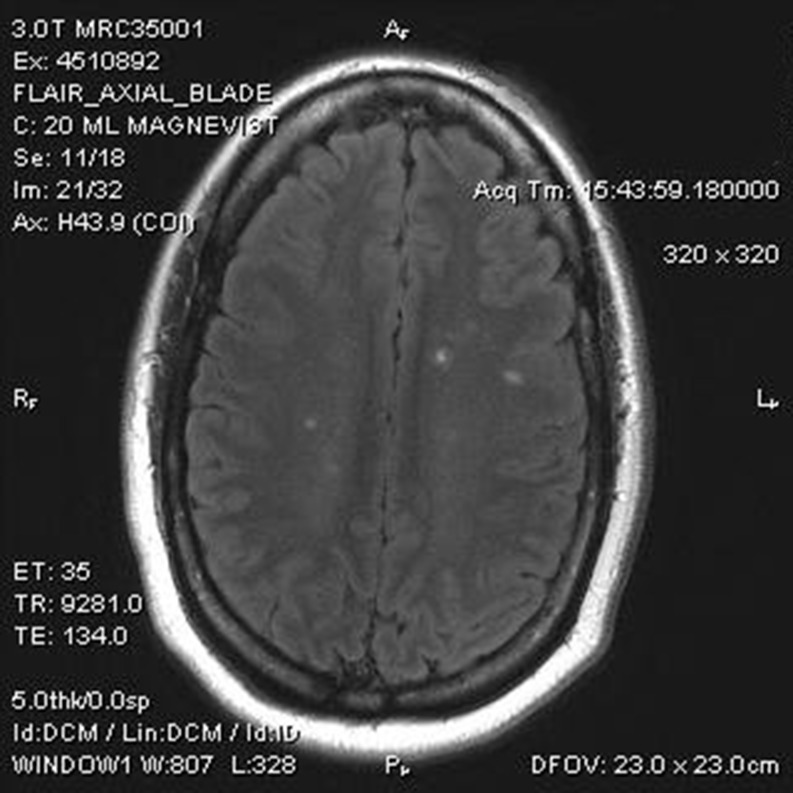
Brain magnetic resonance imaging T2/FLAIR image

Biweekly adalimumab 40 mg injections were initiated because of persistent inflammation of her right eye, despite being treated with steroid-sparing agents, methotrexate then mycophenalote mofetil. Four days after receiving her second injection, she presented with “fuzzy vision” of her right eye. Her visual acuity was reduced to 20/50 with a small right relative afferent pupillary defect, dyschromatopsia, and no pain with movement. Her fundus examination revealed an optic nerve without edema or hemorrhage. Formal visual field testing demonstrated a right superior altitudinal field defect. A brain and orbital magnetic resonance imaging (MRI) with gadolinium showed multiple callosal, pericallosal, periventricular, subcortical, right cerebellar lobe, and left occipital lobe T2/FLAIR lesions with enhancement and restricted diffusion (Fig. 1). Her vision declined to counting fingers and she received methylprednisolone 1 g/day for 3 days followed by a tapering course of prednisone. A lumbar puncture was performed; the CSF was clear and positive for oligoclonal bands. IgG index was elevated to 13.7 (0.8–7.7). Further laboratory evaluation was negative for HTLV I/II, HIV, and HCV antibodies, HBcAg and sAg, Lyme titers and non-reactive to RPR. Her extended review of systems and the remainder of her neurologic examination were unremarkable.

Within a few weeks, she experienced complete resolution and subsequent follow-up visual fields have been full. Six months afterwards, repeat imaging demonstrated no significant interval change in the multiple T2/FLAIR foci of hyperintensities. Several of the lesions still had faint restricted diffusion. Our patient has remained symptom free and has started on immunomodulatory therapy.

## Discussion

Since the introduction of TNFα antagonists into clinical use in 1998, three TNFα antagonists have been approved: two recombinant human monoclonal antibodies adalimumab and infliximab and a soluble receptor etanercept. TNFα antagonists have been found to be efficacious in the treatment of many immune mediated inflammatory diseases, including rheumatoid arthritis, polyarticular juvenile rheumatoid arthritis, Crohn's disease, psoriatic arthritis, and ankylosing spondylitis.

TNFα is a cytokine secreted by T-cells and macrophages that is an important component in the immune-mediation of demyelination. In vivo and in vitro experiments have suggested a correlative relationship between elevated TNFα and the severity of demyelinating disease [5]. The effect of TNF inhibition was investigated in murine experimental autoimmune encephalitis (EAE) and the initial results were promising [6–9]. However, neutralization of TNFα in humans did not result in an improvement in demyelinative disease in multiple sclerosis, but rather it seemed to exacerbate it [10, 11]. Several explanations of its failure in humans include the inability of the TNFα inhibitor to penetrate the blood brain barrier and the complex role that TNFα plays in remyelination and regulation of other cytokines and lymphocytes [12].

Chung et al. [1] first described two cases of adalimumab associated optic neuritis. Subsequently, Simsek et al. [13] reviewed 15 cases of all TNFα antagonist associated optic neuritis in the literature. The interval from the initial administration to presentation ranged from 2 months to 1.5 years (median 7.5 months). All but one were treated with pulse steroids followed by oral steroids. Nine of the 15 patients had a complete visual recovery. Of the eight patients who had a brain MRI reported, only two had findings suggestive of demyelination. After 4 to 6 months, the two with significant MRI findings had only partial visual recovery.

## Conclusion

It has been postulated that TNFα antagonists may induce demyelinating events or even unmask an underlying demyelinating process in those who are predisposed. Our patient with her short temporal proximity to therapy initiation and full recovery without recurrence would suggest a causal association. The large burden of radiographic disease at presentation so quickly after treatment initiation and its persistence would give credence to the theory that an underlying demyelinating process was unmasked by adalimumab.

The occurrence of a demyelinating event with TNFα antagonist treatment is rare; however, the clinician should be aware of the need to observe their patients closely for any manifestations and to institute appropriate therapy.
